# Progress in the Self-Regulation System in Legume Nodule Development-AON (Autoregulation of Nodulation)

**DOI:** 10.3390/ijms23126676

**Published:** 2022-06-15

**Authors:** Yuhe Li, Yue Pei, Yitong Shen, Rui Zhang, Mingming Kang, Yelin Ma, Dengyao Li, Yuhui Chen

**Affiliations:** Ministry of Education Key Laboratory of Cell Activities and Stress Adaptations, School of Life Sciences, Lanzhou University, Lanzhou 730000, China; yhli2019@lzu.edu.cn (Y.L.); peiy20@lzu.edu.cn (Y.P.); shenyt19@lzu.edu.cn (Y.S.); zhangr20@lzu.edu.cn (R.Z.); kangmm21@lzu.edu.cn (M.K.); mayl21@lzu.edu.cn (Y.M.); lidy2018@lzu.edu.cn (D.L.)

**Keywords:** AON, nodule development, legumes, phytohormones, nitrogen nutrient

## Abstract

The formation and development of legumes nodules requires a lot of energy. Legumes must strictly control the number and activity of nodules to ensure efficient energy distribution. The AON system can limit the number of rhizobia infections and nodule numbers through the systemic signal pathway network that the aboveground and belowground parts participate in together. It can also promote the formation of nodules when plants are deficient in nitrogen. The currently known AON pathway includes four parts: soil NO_3_^−^ signal and *Rhizobium* signal recognition and transmission, CLE-SUNN is the negative regulation pathway, CEP-CRA2 is the positive regulation pathway and the miR2111/TML module regulates nodule formation and development. In order to ensure the biological function of this important approach, plants use a variety of plant hormones, polypeptides, receptor kinases, transcription factors and miRNAs for signal transmission and transcriptional regulation. This review summarizes and discusses the research progress of the AON pathway in Legume nodule development.

## 1. Introduction

Legumes are distributed worldwide and are the third largest family of seed plants [1]. They comprise many species, and are an important source of starch, protein, and fat for humans. They can also be used as animal feed. Additionally, the symbiotic nitrogen fixation by legumes and rhizobia is vital for the sustainable development of agriculture. It is also one of the model systems for studying the symbiosis between prokaryotes and eukaryotes. Understanding the mechanisms regulating legumes symbiotic nitrogen fixation is both scientifically and agriculturally significant. The formation and development of functional nodules in legumes is energy intensive, and up to ~25% of the net photosynthetic products of legumes are used for nodule development [2]. Therefore, legumes must strictly regulate the number and activity of nodules to balance the ratio between (1) carbon invested, and nitrogen gained, and (2) plant growth and nodule growth, to ensure efficient energy partitioning. The nutrient acquisition by plants is regulated by resource availability and metabolic requirements, thus indicating communication between the plant roots and the above-ground parts. They exchange signal molecules and nutrients through the vascular network [3].

Evidence indicating the systemic signal control of nodule formation and activity was first found in the early 1950s. Using nodule removal, grafting, and split-root experiments, Kosslak et al. showed that host plants regulate and limit the number of nodules by integrating the stem and root signals [2,3,4,5,6,7,8,9]. This systemic regulation of nodulation is called autoregulation of nodulation (AON) [10]. The AON system limits both rhizobial infection and the number of root nodules. Recently, research on the AON pathway has become more in-depth. The CLAVATA3/EMBRYO-SURROUNDING REGION RELATED–Hypernodulation and Aberrant Root 1 SUper Numeric Nodules (CLE-HAR1/SUNN) negative regulation of nodulation system in *Lotus japonicus* and *Medicago truncatula* was discovered in 2009 and 2010, whereby the number of nodules was limited when the soil nitrogen supply was sufficient [11,12,13,14]. In 2016, a study by Mohd-Radzman et al. proved that the *M. truncatula* polypeptide C-terminally Encoded Peptide (MtCEP1) secreted by the plant root system when nitrogen-deficient was recognized by the receptor kinase COMPACT ROOT ARCHITECTURE 2 (MtCRA2) in the shoot bud, which promoted nodule formation [15]. In 2020, a study by Gautrat et al. found that the CLE/CEP pathways together affect the downstream miR2111/TML module to control the number of nodules and maintain the delicate plant energy balance [16]. The nitrate (NO_3_^−^) signals impact the secretion of CLE and CEP peptides. In 2018, Tsikou and other studies found that high soil NO_3_^−^ concentration can inhibit rhizobial infection and legume nodulation, since NO_3_^−^ uptake from soil is more cost-effective for plants than nitrogen-fixation via symbiosis with rhizobia, as a source of nitrogen [5]. The AON system also inhibits rhizobial infection by weakening the plant’s perception of the rhizobia signals [5,6,7]. Therefore, we have expanded the current research on the AON system to include NO_3_^−^ signaling, as it inhibits nodule initiation and development.

The AON system involves local regulation in roots and long-distance regulation from the root to the aerial part and vice versa and involves important transcription factors such as plant hormones and NIN-mediated identification of rhizobia and NO_3_^−^, the SUNN negative regulatory pathway, the CEP-CRA2 positive regulatory pathway, and, finally, the miR2111/TML regulation of nodule formation and development. The present article summarizes the progress made in research on the AON system, including some of the main genes, proteins, and their mechanisms of action.

## 2. Phytohormones and Transcription Factors Mediate the Recognition of and Response to Rhizobia and the NO_3_^−^ Signals

There is a symbiotic relationship between rhizobia and legume plants that forms via mutual recognition. Flavonoids secreted by the plant roots and the rhizobial nodulation factors results in Ca^2+^ oscillations in the plant cell nucleus, which are recognized by the Ca^2+^-calmodulin-dependent protein kinase, which helps phosphorylate the corresponding transcription factors, and subsequently affects the expression of specific genes, thereby ultimately resulting in nodule formation. The AON pathway controls the nodulation process according to soil rhizobia abundance and soil nitrogen content and also maintains the energy balance in the plant. Therefore, the AON pathway is inseparable from the recognition of and response to rhizobial and the NO_3_^−^ signals. Ethylene (ET), cytokinin (CK), gibberellin (GA), and other plant hormones, along with the transcription factors, mediate the recognition of and response to these signals, together forming a complex regulatory network [17].

The mutual recognition between rhizobia and legumes is necessary for nodule formation, with each of them releasing specific chemical signals into the rhizosphere [18]. Legume plants release flavonoids and activate soil rhizobia to produce nod factors (NFs). NFs are lipochitosanoligosaccharides [19], which are perceived by Nod Factor Receptor 1 (LjNFR1) and LjNFR5 as signal molecules in the *L. japonicus* root [20] and combine with the Symbiotic Receptor-like Kinase (SYMRK) on plasma membrane [21]. The signal from SYMRK is transmitted to the nucleus, causing Ca^2+^ oscillation [18,22,23,24]. These Ca^2+^ oscillations are recognized by the Calcium- and Calmodulin-dependent Protein Kinase Does not Make Infections 3 (CCaMK/DMI3) [25,26] that mediates the phosphorylation of the transcription factor CYCLOPS. Phosphorylated CYCLOPS activates the expression of *Nodule Inception* (*NIN*) [27,28,29], thereby resulting in nodule formation.

NIN is a very important transcription factor in the process of root nodule formation and development and is important in the endodermis cell division (root nodule primordia initiation) and infection thread formation (tubular structure in root cells that promotes rhizobia colonization) [30], which are necessary for nodule formation. The *nin* mutant has defective bacterial identification and shows the phenotype of excessive root hair curling and no nodule formation [27]. *NIN* encodes a transcription factor containing the RWP-RK domain, which participates in many signal regulation pathways at different stages of nodule development [31].

The root nodule organogenesis and the infection thread formation are regulated by plant hormone ET [32,33,34]. It was found that when legume plants were exposed to ET gas or ET precursors (such as ACC), the rhizobial infection and the cell division capacity of the root nodule primordia were inhibited and showed a reduced number of root nodules [33,35,36]. The *L. japonicus ethylene receptor transgenics* (*Ljetr*) and *M. truncatula ethylene insensitive 2* (*Mtein2*) mutant plants, which are important elements in the ET signaling pathway, showed increasing nodule numbers. These findings show that ET inhibits nodule formation [37]. Cerri et al. (2016) found that the Ethylene Responsive Factor (ERF) transcription factor Ethylene Response Factor1 (ERN1) required for nodulation was necessary for nodulation, and the *ern1* mutant could not form nodules, but showed symbiotic reaction in the early stage of rhizobial infection. *MtERN1* is induced by the CCaMK/CYCLOPS complex and controls the expression of the cell wall-related *Early Nodulin 11* (*MtENOD11*) and *MtENOD12* [38,39,40,41], whereas *MtNIN* regulates the degree of infection by restricting the expression of *MtENOD11* in root epidermis, which antagonizes ERN1 and plays a negative regulatory role in rhizobial infection.

**Figure 1:**
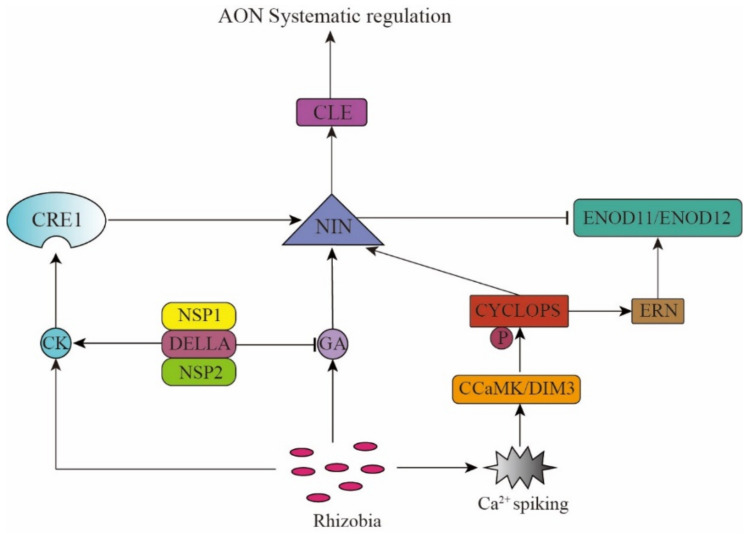
Phytohormones and the transcription factor NIN mediate the rhizobium signal recognition.

In the AON pathway, CK, GA, and NIN help identify the rhizobial and nitrate (NO_3_^−^) signals and trigger the generation of the CLE and CEP system signals. On the one hand, the rhizobia signal promotes the CK synthesis. The CK receptor *CRE1* recognizes the CK signal and promotes the *NIN* gene expression. The transcription factor, NIN, then binds to the promoters of *MtCLE13* and *LjCLE-RS1/2* (*LjCLE-Root Signal1/2*), triggering their expression [12,42,43,44,45,46,47]. On the other hand, rhizobia can also promote *MtCEP7* expression via the *CK/CRE1* and *NIN* pathway and rely on *MtCRA2* to promote nodulation [48]. Hence, the positive or negative regulation of nodulation depends on the changes in the environment and the plant’s needs. The nitrate (NO_3_^−^) signal can also trigger the CLE signal transduction by affecting the activity of transcription factor NIN-Like Protein (NLP) [49,50,51,52], thus indicating that NIN activates the *CLE* gene expression. It is speculated that the CLE peptide inhibits the *NIN* expression via negative feedback regulation, thus promoting nodulation in plants when they are nitrogen deficient, while inhibiting it when nitrogen is sufficient, thereby maintaining the dynamic and delicate balance of plant energy partitioning (Figure 1).

Akamatsu and other researchers found that the GA signal plays a key regulatory role in the AON pathway and inhibits nodule formation. In the process of nodule formation, GA biosynthesis in the vascular bundle of the nodule is activated, causing its accumulation in the nodule. The *NIN* promoter contains a GA-responsive *cis*-acting element, which activates the *NIN* in the vascular bundle and stimulates the differentiation of the nodule cells and inhibits the rhizobial infection. The cis-acting deletion mutant of *NIN* is more susceptible to rhizobial infection, with the GA-inducible expression of CLE-RS1 and CLE-RS2 in the mutant being reduced. This result indicates that the GA inhibition happens through the AON pathway, where GA induces NIN to activate the AON system to regulate nodule formation [53,54]. Mayoshik et al. found that when a high concentration of GA_3_ or GA_4_ (10^−7^–10^−4^ M) were used to treat the roots of *L. japonicus* and other leguminous plants without rhizobia, the cells in the pericycle would divide and form nodule-like structures [53]. However, due to the trace amounts of phytohormones in plants and the multiple functions of GAs, the exact role of GA has not yet been clarified. Fonouni-Farde and others found that the DELLA protein (a negative regulator of GA signal) activates the CK signal in the cortex by regulating the effects of the Nodulation Signaling Pathway 2 (NSP2) and Nuclear Factor-Y 1 (NF-YA1), thereby promoting nodule formation [55,56]. The transcription factors CYCLOPS, NSP1, and NSP2 are functionally conserved in legumes, non-legumes, and mycorrhizal symbiosis. They interact with the DELLA protein to induce the NIN expression in symbiotic nitrogen fixation [57,58]. In peas, DELLA promotes the rhizobial infection of the epidermal cells [59] (Figure 1).

## 3. CLE-SUNN Negatively Regulates the Nodulation Signaling Pathway

**Figure 2:**
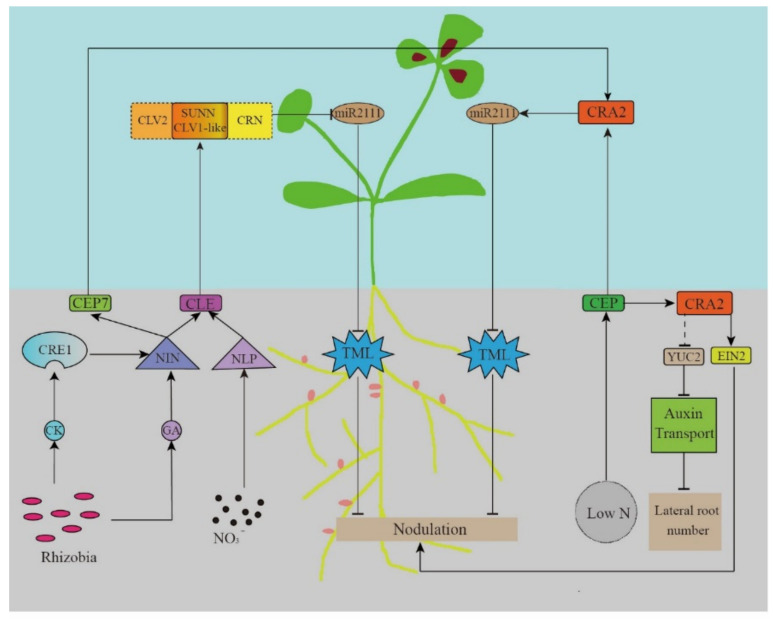
The AON systemic regulatory pathway model.

The long-distance signal transmission of the AON system starts with the secretion of specific peptides. CLE is a small 12-amino acid-long highly-conserved peptide in legumes. Rhizobia induce its expression in the roots, which depends on the soil nitrogen (NO_3_^−^) concentration. Then, it is transported to the above-ground parts through the xylem. CLE is recognized by the receptor kinases Nodule Autoregulation Receptor Kinase (GmNARK)/LjHAR1/MtSUNN, and negatively regulates the nodule formation (Figure 2).

The CLE peptide involved in the AON pathway in *M. truncatula* is encoded by the *MtCLE12*, *MtCLE13*, and *MtCLE35* genes induced by the rhizobial signal [11,12,51,60]. Plants overexpressing *MtCLE12* and *MtCLE13* lose their nodulation ability [13,61]. In *L. japonicus* roots, CLE is encoded by *LjCLE-RS1* (RS1), *LjCLE-RS2*, and *LjCLE-RS3* (*LjCLE-RS2* and *LjCLE-RS3* can also be induced by the NO_3_^−^ signal) [13,62]. Similarly, in soybeans, CLE is encoded by the *GmRIC1* and *GmRIC2* genes (*GmCLE1* can also be induced by the NO_3_^−^ signal) [14].

The stability and activity of polypeptides depend on post-translational modifications. Studies have shown that MtCLE12 is hydroxylated and triarabinosylated by Root Determined Nodation 1 (MtRDN1) in the root system [61,63,64], whereas LjCLE-RS2 and LjCLE-RS3 also undergo the same modifications by LjPLENTY [65,66,67]. Only the modified CLE peptide can inhibit nodule formation. The hydroxyproline arabinosyltransferase (HPAT) in the *rdn1* mutant is damaged, thus showing hyper nodulation phenotype [68,69,70]. CLE can be detected in the xylem sap, suggesting the existence of a long-distance signaling pathway from the roots to the above-ground parts. Follow-up studies have found that CLE in the above-ground parts is regulated by the receptor kinase MtSUNN, LjHAR1 [66,68,69,70,71], or GmNARK, thus influencing signal recognition [14,72].

*GmNARK*/*LjHAR1*/*MtSUNN* encodes a class XI leucine-rich repeats receptor-like kinases (LRR-RLKs) located in the shoot buds, including the N-terminal cell membrane targeting signals, leucine-rich repetitive sequences, the transmembrane domain, and the cytoplasmic serine/threonine kinase domain that are homologous to the *Arabidopsis thaliana* gene *CLAVATA1* (*CLV1*) involved in meristem maintenance [4,68,72,73]. Krusell et al. (2011) found that LjHAR1 (CLV1) interacts with LjCLV2 and LjCRN. The Tnt1 insertion mutant of *MtCRN* has more nodules, and the *CLV2* mutant in pea and *L. japonicus* also shares a similar phenotype [74,75]. In *Arabidopsis thaliana*, a CLAVATA (CLV)-WUSCHEL (WUS) negative feedback loop is important in maintaining the proliferation and differentiation of the stem cells and the continuous initiation and growth of plant organs. Among them, *WUSCHEL* (*WUS*) is necessary for stem cell recognition, whereas the *CLAVATA1*, *2*, and *3* (*CLV*) genes promote organ initiation. The *CLV* gene inhibits the *WUS* gene expression. WUS is a homeodomain transcription factor that directly activates the *CLV3* expression of and promotes the activity of stem cells in the shoot apex meristem. These key regulatory factors interact with each other to establish a negative feedback pathway in the plant shoot apex meristem [76]. Subsequent studies found that a series of receptor kinases either participated in the CLV-WUS pathway independently or formed isoforms. RECEPTOR-LIKE PROTEIN KINASE 2 (RPK2), CLV2, and pseudokinase CORYNE (CRN) participate in the perception of CLV3 signals to inhibit *WUS* expression [77,78]. Similar to the *Arabidopsis* CLV1, LjHAR1, LjCLV2, and LjCRN1 could also form isoforms, with an interaction between LjCRN and LjCLV2. Therefore, we speculate that CLV2 and CRN may play a role in the AON system by forming a multi-protein complex with CLV1 in the legumes as a co-receptor of CLE [74,75].

## 4. CEP-CRA2 Positively Regulates the Nodulation Signal Pathway

A CEP-CRA2 signal opposite to the CLE-SUNN signal also exists in the AON pathway. In the absence of rhizobia or plant nitrogen deficiency, the CEP1 produced by the root system is transported through the xylem and recognized by the receptor CRA2 in the shoot buds to promote nodulation [79,80].

CEP has been proven to be a peptide hormone that is important in regulating the nitrogen (N) demand signals, nodulation, and lateral root development [81,82,83]. Being a PTM (precursor derived, post translationally modified) peptide [84], the *CEP* gene encodes a non-functional peptide that is subsequently processed to produce smaller functional peptides. CEP has an N-terminal signal sequence, one or more conserved CEP domain, and one or more flanking variable region [85]. The proline residues in CEPS are usually hydroxylated, thereby influencing their biological activities [79,86,87,88,89,90]. There is also evidence that the hydroxyproline residues in some CEP peptides have been triacetylated [86]. Despite hydroxylation and triacetylation being well-known for their importance in the activity of CLE peptides, very few studies can be found on how CEP modification affects its activity and nodulation functions (Figure 2).

In *A. thaliana*, *AtCEP1* is expressed under nitrogen deficiency. Using the long-distance root-stem signal transmission, AtCEP1 is recognized by its receptor CEPR1 (CEP Receptor 1) [91]. AtCEP1 affects the transcription of the nitrate transporters NRT1.1, NRT2.1, and NRT3.1, thereby regulating the nitrogen uptake and nitrogen status [90,91,92,93,94,95]. Ohkubo et al. found that the CEP-CEPR1 signal affected the NRT2.1 transcription, contingent on the transport of CEPD1 (CEP Downstream 1) and CEPD2 from the phloem to the roots. Interestingly, CEPD only affected transcription of NRT2.1, but not of NRT1.1 and NRT3.1 [92,94].

In 2016, Radzman et al. demonstrated that the peptide MtCEP1 secreted by roots positively regulates nodulation through the receptor kinase MtCRA2 in shoot buds [15]. MtCRA2 is homologous to *A. thaliana* CEPR1, belonging to the LRR-RLKs receptor kinase family, which participates in the AON pathway as a CEP receptor. Unlike the CEP-CEPR signal in *A. thaliana*, the CEP-CRA2 signal depends on miRNA transmission to the root (see the next section). CRA2 was also expressed in roots and nodules similarly to MtSUNN, a receptor kinase in CLE-SUNN signaling. The study found that compared with wild-type plants, the *cra*2 mutants showed shorter taproots, more lateral roots, and fewer nodules, thereby indicating that CRA2 negatively regulates lateral root formation, while positively regulating the nodule development of [72]. The by CRA2 regulation of the development of lateral roots is independent from that in nodule formation. The regulation of lateral root development is local. Contrastingly, regulating nodule development involves not only the systemic AON pathway, but also the CRA2 expressed in the root. In the roots, MtCRA2 inhibited the MtYCU2 expression and the auxin biosynthesis via unknown pathways, thus regulating the formation of lateral roots. Under nitrogen-deficient conditions, MtCEP1 can also promote MtCRA2 activity and phosphorylate MtEIN2 in roots, which partly relieves the ET inhibition of rhizobial infection and the promotion of nodulation [96].

Taken together, the CEP-CRA2 and CLE-SUNN signals are opposing and independent. They jointly improve the legume response to the soil rhizobia and nitrogen signals and are the key components of the AON pathway, which helps regulate nodule formation and energy balance [97].

## 5. miR2111/TML Responds to the AON Signal to Control Nodulation

As described previously, the plant roots secrete the CLE and CEP peptides to inhibit and promote nodulation, respectively. Both these peptides were transported by xylem and recognized by the receptor kinases SUNN and CRA2, respectively, in the above-ground plant parts. These signals will finally regulate the formation and development of nodules. After the signal is recognized by the receptors in the above-ground plant parts, new signals are produced and transmitted to the roots, thereby strengthening the response to the final effector and directly regulating nodulation [5,6].

The miR2111/TML regulatory module is a common effector of the CLE and CEP signals, which are critical for regulating both root nodulation and development in the AON pathway. Gautrat et al. found that miR2111 in shoot buds transmits signals via the phloem to negatively regulate *TML* expression in the roots [16]. TML was first found in *Lotus* roots, with the *Ljtml* mutant showing a super-nodulation phenotype [98]. Therefore, TML inhibits nodule formation, whereas miR2111 promotes nodule formation [99]. Under nitrogen-deficient conditions, the CRA2 recognizes the CEP1 signal to induce miR2111 and promotes nodule formation. When nitrogen is sufficient and rhizobia is abundant, the CLE-SUNN signal negatively regulates the miR2111 expression and inhibits nodulation [16] (Figure 2). However, the mechanism of the negative regulatory ability of TML is still unclear.

There are two copies of the *TML* gene in *M. truncatula*, *MtTML1* and *MtTML2* [6]. Sequence analysis revealed that the TML protein had two conserved domains (F-box and kelch repeat). The F-box domain that binds to the ASK family proteins to form the Skp1/cul1/F-box (SCF)-E3 ubiquitin ligase complex, while the kelch repeats domain is involved in protein–protein interaction. In the F-box protein containing the kelch repeats, the latter is necessary for physically interacting with its target protein. Therefore, we speculate that the TML protein may regulate nodulation by mediating the degradation of the downstream target proteins.

*A. thaliana* also possesses the miR2111/TML regulatory module, thereby indicating that it is evolutionarily conserved. Besides regulating nodulation, it may play a complementary role in regulating nitrogen utilization. In *Arabidopsis thaliana*, the *MtTML* homologous gene *At3g27150* is also targeted and regulated by miR2111, which can be detected in *Arabidopsis* phloem sap [100]. Interestingly, when compared with legumes, it was phosphate (Pi) deficiency that induced miR2111 in *A. thaliana*, and not nitrogen deficiency [100]. Similarly, the E3 ubiquitin ligase Nitrogen Limitation Attachment (NLA) and E2 ubiquitin binding enzyme PHOSPHATE2 (PHO2) in *A. thaliana* were both negatively regulated by miR827 and miR399 transferred from shoot buds to roots under Pi deficiency, thereby promoting Pi acquisition [101,102,103]. Therefore, these results indicate that the miRNA/F-box regulatory module has multiple functions in the homeostatic regulation of N and P nutrition in higher plants.

## 6. Interplay of AON with Arbuscular Mycorrhizal Symbiosis and Phosphate

Phosphorus is a macronutrient essential for plant growth and development. It participates not only in the biosynthesis of various important organic compounds in plants, but also in various plant metabolic activities in various forms. Legumes, like other land plants, can only absorb phosphorus from the soil as an inorganic phosphate (i.e., orthophosphate; Pi) [104]. However, the soil phosphorus rapidly forms complexes with other ions, thereby making uptake impossible [105]. Most terrestrial plants can form mutualistic symbiosis with arbuscular mycorrhizal (AM) fungi, thereby increasing the plant’s phosphate (Pi) acquisition. The chitin oligomers secreted by AM are recognized by plants, which then activate the fungal symbiosis program via a symbiotic signaling pathway common to rhizobia. Similar to rhizobial symbiosis, AM symbiotic development is systematically regulated by plant Pi levels; high Pi concentrations inhibit both the initiation and further development of symbiosis and also the strigolactone synthesis and its export [106,107]. Since phosphorus promotes the synthesis and export of strigolactones, AM fungal colonization negatively regulates further symbiotic development and is independent of the plant’s phosphorus status [108,109]. This systemic regulation is like AON, and is therefore referred to as the autoregulation of mycorrhization (AOM).

The inhibitory effect of the AOM pathway on AM colonization is also regulated by the system involving the CLE peptide and LRR-RLK CLAVATA1. Mutants of the *CLV1* homologs *GmNARK*, *LjHAR1*, *PvSYM29*, and *MtSUNN* in legumes all showed enhanced AM colonization [110]. Müller and Le Marquer et al. examined the transcript levels of *CLEs* in the AM-colonized roots of *M. truncatula* and showed that the expressions of *MtCLE16*, *MtCLE45*, and *MtCLE53* were induced by AM, with *MtCLE45* and *MtCLE53* expression increasing the most [111,112]. Further studies found that *MtCLE53* expression was induced in AM-colonized roots, which triggered a negative feedback loop that negatively affected colonization by reducing strigolactone levels. Although this effect depends on MtSUNN, other unknown receptors may also be involved [111]. As an early symbiotic signal of AM colonization, strigolactone promotes AM symbiosis, fungal energy metabolism, hyphal bifurcation and the secretion of fungal chitin oligomers, thereby activating the plant symbiotic signaling pathway [18,113]. Moreover, the strigolactone release is also partially regulated by Pi [106], which can directly affect the secretion of fungal chitin oligomers [113,114,115,116].

Additionally, multiple studies have found that Pi deficiency can not only significantly reduce the number of nodules in legumes, but also reduce their nitrogen fixation ability [117,118,119,120]. Physiological studies on different legumes have shown that phosphorus is preferentially transferred from other organs to the root nodules during Pi deficiency [117,119,120]. Isidra-Arellano et al. (2018) showed that Pi deficiency negatively affects early symbiotic events, i.e., the expression of symbiosis-related genes and infection thread formation [121]. The latest study found that the inhibitory effect of Pi deficiency on legume nodulation depends on the AON pathway. Isidra-Arellano et al. (2020) proposed a model for activating the AON pathway under Pi deficiency. During Pi absence, the master regulator of Pi perception and signal transduction, the MYB transcription factor PHR1 (PHOSPHATE STARVATION1), directly binds to NIN and the promoters of *RIC1* and *RIC2*, which encode CLE peptides. These two AON-related CLE peptides are transported from the root to the stem. This activates the negative regulatory pathway mediated by CLE and its receptors in the AON pathway, thereby inhibiting nodulation. Furthermore, PHR1 can also directly bind to the *TML* promoter and promote its expression, thereby reducing nodule growth [122].

## 7. Future Perspectives

The formation of legume symbiotic nodules requires abundant energy. When the soil NO_3_^−^ content is sufficient, it is obviously more economical to absorb nitrogen directly from the soil rather than from symbiotic nitrogen fixation. Therefore, legumes need to control the number of nodules for maintaining the energy and carbon-nitrogen balance.

The nodule number and its development in legumes are strictly limited by the negative feedback regulation of long-distance signals from the roots to shoot buds and back to roots, which is called nodule self-regulation (autoregulation or AON). The AON pathway involves systemic regulation in the root–soil–shoot continuum, including the CLE-SUNN negative regulatory pathway and the CEP-CRA2 positive regulatory pathway. When nitrogen and rhizobia were abundant in the soil, the rhizobial signal can affect the CK concentration, and CRE1 and NIN can trigger the CLE signal transduction. NO_3_^−^ can also trigger the CLE signal transduction by affecting the activity of the NLP transcription factor. When plants are deficient in nitrogen, the rhizobial signal promotes the expression of MtCEP7 through CK/CRE1 and NIN signaling. After the CLE and CEP signals are recognized by SUNN and CRA2, respectively, the downstream signal from the shoot buds to the roots is conducted by the shoot bud-produced miR2111. Activated SUNN and CRA2 inhibit and promote the formation of miR2111, respectively, and then miR2111 negatively regulates the *TML* expression in the roots. This systemic regulatory pathway allows the nodulation capacity of the legume roots to be fine-tuned, depending on the nitrogen utilization.

Existing studies have shown that the understanding of the recognition of rhizobia in the early stage of nodule formation is basically complete. Transcription factors such as NIN and NLP, and plant hormones such as ET, CK, GA, and auxin have an important role in transcriptional regulation and signal transduction. However, some mechanisms in the AON pathway are still unclear.

SUNN is the receptor of the CLE-SUNN system, which is homologous to the CLV1 in *A. thaliana*. In *A. thaliana*, the CLAVATA (CLV)-WUSCHEL (WUS) negative feedback loop is important in maintaining the proliferation and differentiation of stem cells and the continuous initiation and growth of plant organs, including the participation of a series of receptors such as kinases, including CLV1, 2, 3, CRN, and CIKs. 1. Do other receptor-like kinases involved in this pathway also have functions similar to CLV1 in nodulation regulation in legumes? 2. Does WUS also regulate nodule formation and/or development? 3. Given that ClV1, CLV2, and CRN can form homotypic and heteromorphic complexes between each other (whereby CRN, as a pseudokinase, needs to bind with other receptor kinases to play its role), can CLV2 or CLV2-CRN regulate the formation and development of nodules independently of CLV1?

In the CEP-CRA2 signaling pathway, CEP controls the N-demand signal and lateral root development. Interestingly, in *A. thaliana*, the downstream CEP signal is transmitted from CEPD to the roots, which differs from the miR2111/TML module downstream of the AON pathway. Additionally, the miR2111 and TML homologs in *Arabidopsis* affect Pi uptake. If CEPD exists in legumes, what kind of function does it have? Does CRA2 also have a CEPR1/CEPR2 co-receptor? It should be borne in mind that CRA2 also has a relatively independent function in legume roots, i.e., to suppress YUC2 expression. However, the mechanism by which it affects auxin biosynthesis and inhibits lateral root growth still remains unclear.

Downstream of the AON pathway, the miR2111/TML module acts as the common effector of CLE-SUNN and CEP-CRA2 to regulate the formation and development of nodules. However, the mechanism of negative regulation of nodules by TML is still unclear. As an F-box protein containing the kelch repeats domain, TML can theoretically form the Skp1/CUL1/F-box (SCF) E3 ubiquitin ligase complex. In *A. thaliana*, similarly to MtTML, the ZEITLUPE/Flavin-binding Kelch Repeat, F-BOX/LOV Kelch Protein2 (ZTL/FKF1/LKP2) protein family with the F-box and kelch repeats domains can participate in plant photomorphogenesis and stress response by mediating the ubiquitin-mediated proteosomal degradation of specific transcription factors (Timing of CAB Expression1, Cycling Dof Factor 1, etc.) [123]. Recent studies have shown that AtFKF1 can facilitate the ubiquitin-mediated proteosomal degradation of DELLA protein via the kelch repeat domain and the GRAS domain of DELLA protein, thereby promoting the flowering of *Arabidopsis thaliana* under long-day conditions [124]. Does MtTML also negatively regulate nodule formation by mediating DELLA or other transcription factors (such as NLP, NSP, etc.) involved in the process of nodule formation? Moreover, *miR827*/AtNLA, *miR399*/AtPHO2, and miR2111/MtTML have similar functions in *Arabidopsis thaliana*. Are miRNAs, such as *miR827*, *miR399*, etc., also involved in regulating nodule development?

Recently, there have been many studies on the formation and development of legume nodules. As an important regulatory pathway to balance nodule energy consumption with other plant energy demands, the AON pathway is a hot research topic. Summarizing the existing research results, we found that not only many important AON pathway genes have been reported, but also their homologs in *A. thaliana* have similar functions, such as regulating nitrogen and phosphorus uptake and meristem differentiation. This is because the AON pathway is closely related to plant energy partitioning, and the selective meristem differentiation is crucial to the formation of nodules. Due to the existence of nodulation mechanism in legumes, these genes have been endowed with new functions related to regulating nodulation in legumes.

In conclusion, while exploring new genes regulating nodule development, we can also refer to their research results in *A. thaliana* and focus on the genes related to energy partitioning and hormone regulation, thus providing new ideas for further studying the nodulation mechanism of legumes in the future.

## Data Availability

Data available in a publicly accessible repository.
